# Potential associations between behavior change techniques and engagement with mobile health apps: a systematic review

**DOI:** 10.3389/fpsyg.2023.1227443

**Published:** 2023-09-18

**Authors:** Madison Milne-Ives, Sophie R. Homer, Jackie Andrade, Edward Meinert

**Affiliations:** ^1^Centre for Health Technology, University of Plymouth, Plymouth, United Kingdom; ^2^Translational and Clinical Research Institute, Newcastle University, Newcastle upon Tyne, United Kingdom; ^3^School of Psychology, Faculty of Health, University of Plymouth, Plymouth, United Kingdom; ^4^Department of Primary Care and Public Health, School of Public Health, Imperial College London, London, United Kingdom

**Keywords:** engagement, behaviour change techniques, telemedicine, mobile applications, digital health

## Abstract

**Introduction:**

Lack of engagement is a common challenge for digital health interventions. To achieve their potential, it is necessary to understand how best to support users’ engagement with interventions and target health behaviors. The aim of this systematic review was to identify the behavioral theories and behavior change techniques being incorporated into mobile health apps and how they are associated with the different components of engagement.

**Methods:**

The review was structured using the PRISMA and PICOS frameworks and searched six databases in July 2022: PubMed, Embase, CINAHL, APA PsycArticles, ScienceDirect, and Web of Science. Risk of bias was evaluated using the Cochrane Collaboration Risk of Bias 2 and the Mixed Methods Appraisal Tools.

**Analysis:**

A descriptive analysis provided an overview of study and app characteristics and evidence for potential associations between Behavior Change Techniques (BCTs) and engagement was examined.

**Results:**

The final analysis included 28 studies. Six BCTs were repeatedly associated with user engagement: goal setting, self-monitoring of behavior, feedback on behavior, prompts/cues, rewards, and social support. There was insufficient data reported to examine associations with specific components of engagement, but the analysis indicated that the different components were being captured by various measures.

**Conclusion:**

This review provides further evidence supporting the use of common BCTs in mobile health apps. To enable developers to leverage BCTs and other app features to optimize engagement in specific contexts and individual characteristics, we need a better understanding of how BCTs are associated with different components of engagement.

**Systematic review registration:**

https://www.crd.york.ac.uk/prospero/, identifier CRD42022312596.

## 1. Introduction

Mobile health applications have the potential to empower people to improve their health behaviors and self-manage health conditions (Forman et al., 2016; Moller et al., 2017; Digital Implementation Investment Guide [DIIG], 2020). They are widely available, with a large global market (Grand View Research, 2021), and provide a means of delivering far-reaching behavioral interventions. To have a significant impact on behavioral and health outcomes, mobile health apps need to be able to support sufficient engagement to achieve the aims of the intervention they are delivering (Yardley et al., 2016; Cole-Lewis et al., 2019). Previous research has established an association between engagement with digital interventions and their impact on intended outcomes (Perski et al., 2017; Grady et al., 2018; Mclaughlin et al., 2021), demonstrating the importance of a certain degree of engagement for the efficacy of an intervention. Maintaining engagement is a common challenge for mobile health apps (Birnbaum et al., 2015; Yeager and Benight, 2018; Baumel et al., 2019; Meyerowitz-Katz et al., 2020; Pratap et al., 2020; Torous et al., 2020a) and is a potential reason why evidence of their effectiveness remains mixed (Dounavi and Tsoumani, 2019; Ng et al., 2019; Milne-Ives et al., 2020; Moshe et al., 2021).

The conceptualization of engagement has recently become a focus in digital health research, resulting in several theories and frameworks (O’Brien, 2016; Yardley et al., 2016; Perski et al., 2017; Cole-Lewis et al., 2019; Kelders et al., 2020). It is generally accepted to be multi-faceted, with components relating to dimension (i.e., affective, cognitive, and behavioral) and scale [i.e., small-scale engagement with specific components of the digital intervention (“micro”) and larger-scale engagement with target health behaviors (“macro”)] (Milne-Ives et al., 2022). A variety of strategies have been proposed and incorporated into mobile health apps to help improve user engagement, including design features and behavior change techniques (Garnett et al., 2015; Floryan et al., 2020; Iribarren et al., 2021).

Although evidence is still developing, previous research has emphasized the importance of incorporating behavioral theory into digital health intervention design (Gourlan et al., 2016; Moller et al., 2017; Roberts et al., 2017; Klonoff, 2019; Taj et al., 2019). Behavioral theory, and behavior change techniques (a taxonomy of the smallest intervention components that can support behavior change, e.g., goal setting, self-monitoring, rewards), can provide evidence-based predictions to optimize context-specific intervention design and potential impact (Michie et al., 2013; Morrison, 2015; Moller et al., 2017). Impact on engagement, however, is often measured using only behavioral measures of app use (Torous et al., 2020b; Mclaughlin et al., 2021), which cannot capture the full picture of users’ multi-faceted engagement. For this reason, our understanding of how different components of engagement are associated with app features, engagement strategies, and outcomes is incomplete.

Despite the growing presence of Behavior Change Techniques (BCTs) (Michie et al., 2013) in mobile health apps, there has been limited investigation of how they can help support engagement specifically. Our preliminary review of the literature (Milne-Ives et al., 2022) identified several systematic reviews that have explored questions around this topic (O’Connor et al., 2016; Szinay et al., 2020; Wei et al., 2020; Borghouts et al., 2021), but none that analyzed how BCTs can best support the various components of engagement. The purpose of this review was to investigate how specific BCTs are associated with the different components of engagement (affective, cognitive, behavioral, micro, and macro) to provide insights for the development of mobile health apps. To examine this, the primary objective was to identify BCTs being incorporated in the design and development of mobile health apps and their hypothesized or evidenced associations with engagement and its various components. Learnings from this review will enable future studies to empirically test causal relationships between specific BCTs and specific components of engagement to further improve our understanding of their associations.

## 2. Methods

### 2.1. Study design

The Preferred Reporting Items for Systematic Reviews and Meta-analysis (PRISMA) framework (Page et al., 2021) was used to structure this review (Supplementary Appendix 1). The protocol was prospectively registered on PROSPERO (CRD42022312596) and published before searches began (Milne-Ives et al., 2022).

### 2.2. Eligibility criteria

The eligibility criteria and search strategy were based on the PICOS framework (Table 1; Richardson et al., 1995; Counsell, 1997). The protocol scope was broadened to include development studies to capture research that hypothesized potential associations. Studies published before 2011 were excluded because we wanted to provide an overview of recent evidence [digital technology evolves rapidly (Steinhubl et al., 2015)] and because the conceptualization of engagement as a multi-faceted construct emerged in digital health relatively recently (Kelders et al., 2020).

**TABLE 1:** PICOS framework.

Component	Inclusion criteria	Exclusion criteria
Population	Mobile health app users of any age (adults and children)	
Intervention	Mobile health apps developed using at least one behavior change theory, framework, or technique to target at least one health behavior (including but not limited to: physical activity, diet, sedentary behavior, substance use, etc).	Studies with no description of behavioral theory or BCT; authors needed to be able to meaningfully identify behaviorally-based features if they were not reported as BCTs
Comparator	No comparator is required	
Outcomes	App engagement (hypothesized or evidenced), defined broadly to include any components of engagement (affective, cognitive, behavioral; relating to the intervention interface, components, or target health behavior) and any type of measurement (quantitative or qualitative). Secondary outcomes will include the BCTs and theories incorporated in the apps, qualitative or quantitative engagement outcomes measured (including any components of engagement specified by a theoretical framework) and the behavioral and health outcomes reported.	
Study types	Studies that describe the design, development, or evaluation of digital health behavior change interventions that aim to support user engagement (including randomized controlled trials, quantitative, qualitative, cohort, and case studies)	Reviews, protocols, and conference abstracts or posters without full texts

### 2.3. Search strategy

PubMed, Embase, Cumulative Index to Nursing and Allied Health Literature (CINAHL), APA PsycArticles, ScienceDirect, and Web of Science were searched using the search structure (Table 2 and Supplementary Appendix 2): engagement (MeSH OR Keywords) AND digital health interventions (MeSH OR Keywords) AND behavior change (MeSH OR Keywords). Searches were conducted on 12 July 2022.

**TABLE 2:** Search terms.

Category	MeSH^a^	Keywords (in full text)
Engagement	Treatment Adherence and Compliance OR Patient Participation OR Patient Compliance	Engag* OR “user engagement” OR immersion OR flow OR involvement OR presence OR adherence OR attrition OR compliance OR maintenance OR acceptability OR satisfaction
Digital health interventions	Telemedicine OR Mobile Applications OR Internet-Based Intervention	“mHealth” OR “eHealth” OR “mobile health” OR telehealth OR mobile OR phone OR smartphone OR cell OR digital OR “app” OR “apps” OR application* OR digital OR web OR internet
Behavior change	Behavior Control OR Psychological Theory	“behavior change techniques” or “behaviour change techniques” or “BCT” or “behavior change technique” or “behaviour change technique” or “behavioral change strategies” or “behavioural change strategies” or “behavior change wheel” or “behaviour change wheel” or “behavioral theory” or “behavioural theory” or “behavior change theory” or “behaviour change theory” or “health behaviour change” or “behavior change” or “behaviour change” or “digital behavior change intervention” or “digital behaviour change intervention” or “DBCI” or “behaviour change intervention”

### 2.4. Screening and article selection

References were imported into EndNote X9 for duplicate removal and initial screening using the EndNote search function (Supplementary Appendix 3). One reviewer screened titles and abstracts of remaining references in Rayyan and then conducted a full-text review.

### 2.5. Data extraction

Data was extracted by one reviewer into a predetermined form (see Box 1).

**BOX 1 Article information and data extraction.**

**General study information**

● Year of publication

● Country of study

● Sample demographics (eg. age, gender, target population)

● Initial sample size

● Analyzed sample size

● Study duration

**Mobile health app behavioral intervention**

● App name

● Operating platform (iOS, Android, web)

● Target health behavior

● Aim of the intervention

● Behavioral theory used

● Number of included Behavior Change Techniques (Michie et al., 2013) (if any)

● List of included Behavior Change Techniques (Michie et al., 2013) (if any)

● Other app engagement features

**Evaluation**

● Component(s) of engagement examined

● Engagement outcome measures

● Effect of intervention on engagement outcomes

● Effect of intervention on behavioral outcomes (if reported)

● Effect of intervention on health outcomes (if reported)

### 2.6. Data analysis

A descriptive analysis summarized study and intervention characteristics. A quantitative meta-analysis of effect was not possible due to outcome variety, so extracted evidence was analyzed by one author to map potential associations between BCTs and engagement. Evidence for potential associations was divided into three categories: evidenced, hypothesized, or inferred. Associations were inferred if studies did not report app features as BCTs or did not directly examine associations between features and engagement. Features were coded using the Behaviour Change Technique (BCT) Taxonomy v1 (Michie et al., 2013) as generically as possible (e.g., “non-specific” reward) to ensure that they could be analyzed in line with the rest of the included studies. To examine components of engagement, a basic codebook was created using definitions from the literature (Supplementary Appendix 4) to explore how they were assessed and associated with BCTs.

### 2.7. Risk of bias and quality assessment

The quality appraisal was conducted using the Cochrane Collaboration Risk of Bias 2 (RoB 2) tool for randomized controlled trials (RCTs) (Higgins et al., 2011; The Cochrane Collaboration, 2021) and the Mixed-Methods Appraisal Tool (MMAT) (Hong et al., 2018). The MMAT tool was used instead of the ROBINS-I tool (Sterne et al., 2016) because it could be applied to the wide range of study types included in the review.

## 3. Results

### 3.1. Included studies

**FIGURE 1:**
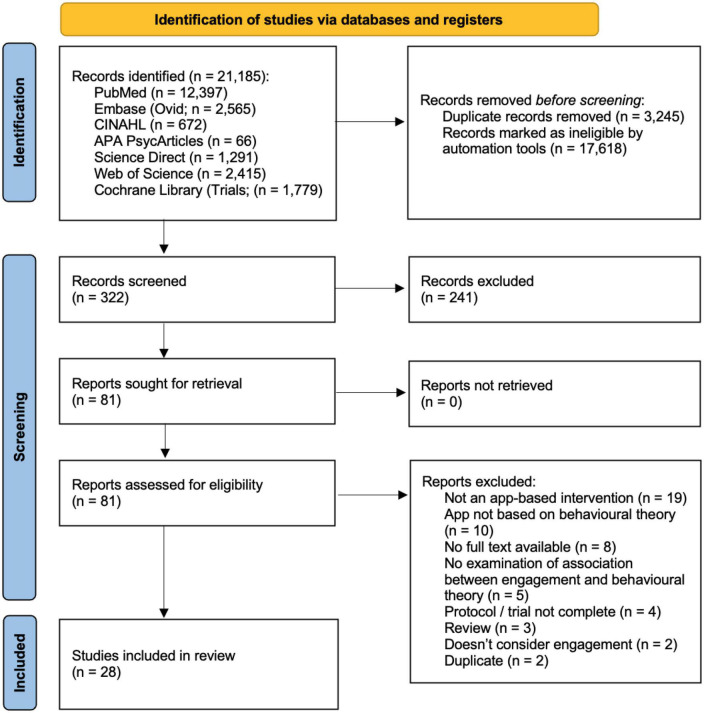
PRISMA flow diagram (Page et al., 2021).

In total, 21,185 articles were retrieved and 28 were determined to be eligible for inclusion. The reasons for exclusion in the full-text review stage are included in the PRISMA flow diagram (Figure 1). One of the included references was a Clinicaltrials.gov registration from which a full text article was identified.

### 3.2. Study characteristics

The characteristics of the 28 included studies are summarized in Supplementary Appendix 5. Qualitative study designs were most common (8/28, 29%). Half of the studies (14/28) described development or early-stage testing. Reported study durations ranged from 2 (Duff et al., 2018) to 83 weeks (Delmas and Kohli, 2020). Sample sizes ranged from 7 (Garnett et al., 2015) to 2,740 (Delmas and Kohli, 2020). Almost three quarters of the studies (18/25) had fewer than 100 participants; the two studies with the largest sample sizes both retrospectively sampled real world app users (Cueto et al., 2019; Delmas and Kohli, 2020). Two thirds of the studies (17/25) focused on adults, a quarter (6/25) on children and adolescents, and two on adolescents and young adults.

### 3.3. App characteristics

Twenty-five individual apps were examined in 25 studies (Table 3), with three studies including multiple apps (Tang et al., 2015; Baumel and Kane, 2018; Szinay et al., 2021). The studies referenced a total of 19 unique behavioral theories (Table 4), with a third (9/28) referencing more than one. Just over half the studies (15/28) explicitly reported BCTs, eight of which did not reference a theory (Garnett et al., 2015; Tang et al., 2015; Cueto et al., 2019; Davis and Ellis, 2019; DeSmet et al., 2019; D’Addario et al., 2020; Valle et al., 2020; Thornton et al., 2021). Among the 28 studies, 40 unique BCTs were identified as included in the apps.

**TABLE 3:** Summary of app characteristics.

References	App name	Operating platform	Target health behavior	Aim of intervention	Behav. theory^a^
Ahonkhai et al., 2021	PEERNaija	Android	Medication adherence	Adherence	SCT
Baumel and Kane, 2018	Many included	Android and web	Several (mental health, diet, physical activity, reduce substance use)	Self-management of health	Not specified
Buman et al., 2016	BeWell24	Android	Sleep, sedentary, and physical activity behaviors	Improve cardiometabolic health outcomes	BCTs, SCT, sleep education, sleep hygiene, stimulus control therapy, self-regulation
Caon et al., 2022	PEGASO Companion (eDiary)	Android	Dietary behaviors	Weight management	COM-B model
Cueto et al., 2019	Kurbo	Not specified	Dietary and physical activity behaviors	Weight management	BCTs
Curtis et al., 2019	MyMate&Me	Not specified	Medication adherence	Adherence	BCW
D’Addario et al., 2020	Not specified	Not specified	Physical activity behavior	Increase physical activity	BCTs
Davis and Ellis, 2019	1) Noom Walk Pedometer (NWP; low BCT) 2) MapMyFitness (MMF; high BCT)	iOS	Physical activity behavior	Increase physical activity	BCTs
Delmas and Kohli, 2020	AirForU	iOS and Android	Air quality related behaviors	Educate about air pollution and protective behaviors	Theory of Issue Engagement, TPB
DeSmet et al., 2019	Not specified	Not specified	Sleep, sedentary, and physical activity behaviors	Increase physical activity (and sleep, and decrease sedentary behavior)	BCTs
Du et al., 2016	Fittle	iOS and Android	Dietary and physical activity behaviors	Adherence (to healthy habits)	TPB, SCT
Duff et al., 2018	MedFit App	Android	Physical activity behaviors	Self-management of health	SCT, BCW
Garnett et al., 2015	Not specified	Not specified	Reduce substance use	Reduce alcohol consumption	BCTs
Höchsmann et al., 2019	Mission: Schweinehund	iOS and Android	Physical activity behavior	Increase physical activity	SDT
Hales et al., 2016	Social Pounds Off Digitally (POD)	Android	Dietary and physical activity behaviors	Weight management	SCT
Lin and Mâsse, 2021	Aim2Be	Not specified	Several (dietary and physical activity behaviors, screen time, sleep)	Adherence (to healthy habits)	SCT, SDT
Nuijten et al., 2021	GameBus	iOS and Android	Several	Adherence (to healthy habits)	Social comparison model of competition
Nuijten et al., 2022	GameBus	iOS and Android	Physical activity behaviors	Increase physical activity	COM-B, Fogg Behavior Model, TPB, SDT, Flow theory^b^
Nurmi et al., 2020	Precious app	Android	Physical activity behaviors	Adherence (to healthy habits)	SDT
Smith et al., 2022	Stay-Active	Not specified	Physical activity behaviors	Increase physical activity	BCW, COM-B model, TDF
Songtaweesin et al., 2021	P3 (Prepared, Protected, emPowered) Thailand app	iOS and Android	Medication adherence	Adherence	SCT, Narrative Comm-unication, Fogg behavioral model
Sporrel et al., 2020	Playful data-driven Active Urban Living (PAUL)	Android	Physical activity behaviors	Increase physical activity	BCW, Behavioral Intervention Technology model
Szinay et al., 2021	Many included	Not specified	Several	Not specified	COM-B model, TDF
Tang et al., 2015	Many included	Not specified	Dietary and physical activity behaviors	Weight management	BCTs
Thornton et al., 2021	Health4Life App	iOS and Android	Several (dietary and physical activity behaviors, reduce substance use)	Adherence (to healthy habits)	BCTs
Valle et al., 2020	Nudge	iOS	Dietary and physical activity behaviors	Weight management	BCTs
Whiteley et al., 2018	Battle Viro	iOS	Medication adherence	Adherence	IMB model
Whiteley et al., 2019	Viral Combat	iOS	Medication adherence	Adherence	IMB model

^a^SCT, Social Cognitive Theory; COM-B, Capability, Opportunity, Motivation - Behavior model; BCW, Behavior Change Wheel; TPB, Theory of Planned Behavior; BCTs, Behavior Change Techniques; SDT, Self-Determination Theory; TDF, Theoretical Domains Framework; IMB model, Information, Motivation, and Behavioral Skills Model.

^b^Discussed in paper, but unclear if incorporated in app design.

**TABLE 4:** Behavioral theories used in apps targeting particular health behaviors.

Target behavior	BCTs only	SCT	BCW^a^	COM-B model^a^	TDF	SDT	TPB	Fogg behav. model	IMB model	Other
Physical activity^b^	8	5	2	2	1	4	2	1	–	4
Dietary behavior	4	3	–	1	–	1	1	–	–	–
Medication adherence	–	2	1	–	–	–	–	1	2	1
Substance use	2	–	–	–	–	–	–	–	–	–
Air quality behavior	–	–	–	–	–	–	1	–	–	1
Several (not specified)	–	–	–	1	1	–	–	–	–	1
*Total*	*14*	*10*	*3*	*4*	*2*	*5*	*4*	*2*	*2*	*7*

^a^The core of the BCW is the COM-B model, but they are reported separately here in line with what the studies reported.

^b^Physical activity included movement behaviors, sedentary behaviors, and sleep.

An occurrence was added each time a behavioral theory was reported in a study for each of the target behaviors supported by that app; therefore, columns and rows do not necessarily add up to the number of studies, as several studies included multiple target behaviors or behavioral theories.

### 3.4. Engagement measures

The most common method of measuring engagement (11/28 studies) was through app usage data (e.g., frequency, duration). Other methods included surveys, qualitative methods, app-collected data (active or passive), and data from synced devices (e.g., activity trackers and smart pill bottle caps, Table 5). Many of the studies that used qualitative methods were focused on user-centered design or early-stage usability or feasibility testing.

**TABLE 5:** Summary of engagement measures used in the studies.

References^a^	Intervention data (freq., duration)	Behav. data self-reported on app	Behav. sensor data (app or synced device)	Survey	Qualitative
Baumel and Kane, 2018	X	–	–	Enlight Quality Ratings (EQR)	–
Buman et al., 2016	X	–	–	–	–
Caon et al., 2022	X	X	–	–	–
Cueto et al., 2019	X	X	–	–	–
D’Addario et al., 2020	–	–	–	–	X
Davis and Ellis, 2019	–	–	–	User Mobile App Rating Scale (uMARS)	–
Delmas and Kohli, 2020	X	X	–	–	–
DeSmet et al., 2019	–	–	–	Validated scales to measure intention- to-change	–
Du et al., 2016	–	X	–	–	–
Duff et al., 2018	–	–	–	–	X
Hales et al., 2016	X	X	–	–	X
Höchsmann et al., 2019	X	–	X	Intrinsic Motivation Inventory	–
Lin and Mâsse, 2021	X	–	–	–	–
Nuijten et al., 2021	X	X	–	–	–
Nuijten et al., 2022	X	X	X	–	–
Nurmi et al., 2020	–	–	–	–	X
Songtaweesin et al., 2021	–	–	–	–	X
Szinay et al., 2021	–	–	–	–	X
Tang et al., 2015	–	–	–	–	X
Thornton et al., 2021	–	–	–	System Usability Scale (SUS)	X
Valle et al., 2020	X	X	X	–	–
Whiteley et al., 2019	–	–	–	Client Service Questionnaire (CSQ)	X
**Total**	**11**	**8**	**3**	**6**	**9**

^a^6 papers are absent from the table, as they did not include engagement measures (Garnett et al., 2015; Whiteley et al., 2018; Curtis et al., 2019; Sporrel et al., 2020; Ahonkhai et al., 2021; Smith et al., 2022).

### 3.5. Associations between behavior change techniques and engagement

Table 6 summarizes the authors’ analysis of the potential associations between BCTs and engagement, in the context of the available descriptions and evidence. Methods in the “evidenced” group of studies included comparisons between different mobile conditions (e.g., with and without social support) (Du et al., 2016; Nuijten et al., 2021, 2022), rankings of the effectiveness of particular BCTs for engagement by experts or users (Garnett et al., 2015; Tang et al., 2015; Szinay et al., 2021), and modeling (Delmas and Kohli, 2020; Lin and Mâsse, 2021). Overall, the “evidenced” studies were of moderate quality: of the randomized trials, two had some risk of bias (Du et al., 2016; Nuijten et al., 2022) and one had high risk of bias (Nuijten et al., 2021), but the studies evaluated using the MMAT met most or all of the quality criteria (Garnett et al., 2015; Tang et al., 2015; Delmas and Kohli, 2020; Lin and Mâsse, 2021; Szinay et al., 2021). The six most common BCTs associated with engagement (based on the summed number of studies from the three categories) were social support, goal setting, feedback, prompts/cues, self-monitoring, and rewards (Table 7).

**TABLE 6:** Analysis of potential associations between BCTs and engagement and the evidence for the association.

References	BCTs inferred or explicit in paper?	Included BCTs with potential associations with engagement	Evidence of association between BCTs and engagement	Evidence and analysis - key points	RoB/ QA^a^
Delmas and Kohli, 2020	Inferred	7.1 Prompts/cues	Evidenced in study	● Notifications were a significant predictor of checking the app (β = 0.890, SE = 0.026, *p* < 0.01) and sharing air quality information with others (β = 0.680, SE = 0.086, *p* < 0.01)	****
Du et al., 2016	Inferred	3.1 Social support (unspecified)	Evidenced in study	● Comparison Team and Solo mobile app conditions found Team condition had significantly higher compliance (*M* = 0.49, SD = 0.35) than Solo condition (*M* = 0.30, SD = 0.39; t 50.82 = 1.94, *p* = 0.05) ● Team condition participants were 66% more likely to engage longer with the app than Solo condition participants	Some concerns (RoB)
Garnett et al., 2015	Explicit in paper	1.1 Goal setting (behavior) 1.4 Action planning 2.2 Feedback on behavior 2.3 Self-monitoring of behavior	Evidenced in study	● Expert consensus process identified top 4 BCTs most likely to reduce alcohol consumption ● Highest ranked engagement strategies were ease of use, design aesthetic, and feedback	****
Lin and Mâsse, 2021	Explicit in paper	1.1 Goal setting (behavior) 1.4 Action planning^b^ 3.1 Social support (unspecified) 5.3 Information about social and environmental consequences^b^ 6.1 Demonstration of the behavior 7.1 Prompts/cues^b^ 8.1 Behavioral practice/rehearsal^b^ 8.3 Habit formation^b^	Evidenced in study	● Factor modeling compared engagement profiles (uninvolved, dabblers, engaged, and keeners) ● App features with the highest engagement across the different profiles were aims (BCT = goal setting), social wall (BCT = social support and demonstration of behaviors), and virtual coach (BCT = social support)	****
Nuijten et al., 2021	Inferred	3.1 Social support (unspecified)	Evidenced in study	● Compared intergroup (between class competition, between class competition with teachers separate) and intragroup conditions (within class competition) ● Significantly more app visits by students in intergroup condition (between classes) than students in intragroup competition (M diff = + 0.469 days, *p* < 0.001) ● Students in intergroup condition (between classes) also completed more activities than other two conditions, but this was not significant	High RoB
Nuijten et al., 2022	Inferred	1.1 Goal setting (behavior)	Evidenced in study	● Participants who set a physical activity goal visited the app significantly more than participants who did not (M diff = + 2.176 days for maintenance goal, *p* < 0.001; + 1.625 days for improvement goal, *p* = 0.005) ● Participants who set a goal also registered significantly more activities than those who did not (M diff = + 1.535 activities for maintenance goal, *p* = 0.03; + 3.258 activities for improvement goal, *p* < 0.001)	Some concerns (RoB)
Szinay et al., 2021	Inferred	1.1 Goal setting (behavior) 1.4 Action planning 2.2 Feedback on behavior 2.3 Self-monitoring of behavior 3.1 Social support (unspecified) 3.2 Social support (practical) 5.1 Information about health consequences 7.1 Prompts/cues 8.3 Habit formation 10.3 Non-specific reward	Evidenced in study	● Factors identified in interviews as most important for participants’ engagement included: knowledge (e.g., user guidance and statistical information), memory, attention, and decision processes (e.g., reduced cognitive load), environmental resources (e.g., tailored technology) and social influences (e.g., peer and professional support)	**** *
Tang et al., 2015	Explicit in paper	1.1 Goal setting (behavior) 1.4 Action planning 2.2 Feedback on behavior 2.3 Self-monitoring of behavior 3.1 Social support (unspecified) 3.2 Social support (practical) 5.5 Anticipated regret 5.6 Information about emotional consequences 7.1 Prompts/cues 10.3 Non-specific reward 10.9 Self-reward	Evidenced in study	● Interviews identified several BCTs that were related to engagement by at least 2 participants, including: goal setting, if-then planning, self-monitoring, feedback, awareness of emotional consequences, symbolic rewards, and social contact/support	**** *
Ahonkhai et al., 2021	Inferred	1.1 Goal setting (behavior) 2.2 Feedback on behavior 2.3 Self-monitoring of behavior 3.1 Social support (unspecified) 6.2 Social comparison 10.3 Non-specific reward 14.2 Punishment	Hypothesized	● Identified key barriers to adherence from literature and interviews: forgetfulness, poor executive functioning, and poor social support ● Hypothesized that goal setting, feedback, rewards, social comparison, social support, gamification, fun/playfulness, avatars will increase engagement	N/A
Curtis et al., 2019	Explicit in paper	1.2 Problem solving 1.3 Goal setting (outcome) 1.4 Action planning 2.2 Feedback on behavior 2.3 Self-monitoring of behavior 3.2 Social support (practical) 3.3 Social support (emotional) 4.2 Information about antecedents 5.1 Information about health consequences 6.1 Demonstration of the behavior 6.2 Social comparison 7.1 Prompts/cues 8.1 behavioral practice/rehearsal 8.3 Habit formation 11.2 Reduce negative emotions 12.5 Adding objects to the environment	Hypothesized	● Identified key barriers to adherence from literature and interviews: capability (remembering), motivation (beliefs in effectiveness, confidence in self-managing), opportunity (limited time, changing social support) ● Theoretical behavioral analysis (COM-B and TDF) used to propose BCTs to address barriers and support medication adherence	**** *
D’Addario et al., 2020	Explicit in paper	1.1 Goal setting (behavior) 2.2 Feedback on behavior 2.3 Self-monitoring of behavior 3.1 Social support (unspecified) 6.2 Social comparison^b^ (***NEGATIVE***)	Hypothesized	● Focus groups identified preferences for autonomy and self-regulation features: suggests BCTs such as goal setting, feedback, and self-monitoring are associated with higher engagement ● Social support and self-comparison were perceived as important for motivation ● Participants did not want competition and social comparison for physical activity	**** *
Duff et al., 2018	Explicit in paper	1.1 Goal setting (behavior) 2.3 Self-monitoring of behavior 3.2 Social support (practical) 5.1 Information about health consequences	Hypothesized	● Identified most frequently used BCTs from a systematic review and incorporated in app design ● Focus groups indicated that participants wanted to monitor progress, felt prompts and cues would support awareness and motivation, and would like social interaction	**** *
Hales et al., 2016	Inferred	2.2 Feedback on behavior 3.1 Social support (unspecified) 3.3 Social support (emotional) 4.1 Instruction to perform the behavior 6.2 Social comparison 7.1 Prompts/cues 10.2 Material reward 10.3 Non-specific reward	Hypothesized	● Key themes from focus groups indicated that participants wanted to see others’ progress and send them encouragement, found notifications and podcast information helpful, and suggested adding an incentive system and increasing praise/positive feedback	N/A
Smith et al., 2022	Explicit in paper	1.1 Goal setting 1.4 Action planning 1.5 Review behavior goals 2.2 Feedback on behavior 2.3 Self-monitoring of behavior 3.3 Social support (emotional) 4.1 Instruction to perform the behavior 5.1 Information about health consequences 7.1 Prompts/cues 9.1 Credible source 15.1 Written persuasion about capabilities	Hypothesized	● Focus groups with key stakeholders identified the most appropriate BCTs to achieve engagement with behavior (physical activity) ● Hypothesize that motivational interviewing (social support) and personalized reminders (prompts/cues) will support engagement with intervention	****
Songtaweesin et al., 2021	Inferred	7.1 Prompts/cues 10.2 Material reward 10.3 Non-specific reward	Hypothesized	● Focus groups and interviews identified recommendations for improving app engagement: dynamic content, notifications, fun features, earning redeemable points, and choose-your-own adventure storytelling	**** *
Sporrel et al., 2020	Inferred	1.1 Goal setting (behavior) 2.2 Feedback on behavior 2.3 Self-monitoring of behavior 4.1 Instruction to perform the behavior 5.6 Information about emotional consequences 7.1 Prompts/cues 10.3 Non-specific reward	Hypothesized	● Focus groups and literature review identified preferences for reminders, feedback about goal progress, messages focusing on positive emotional outcomes, personalisable goals, and instruction videos ● Social strategies not necessarily associated with increased motivation to engage in physical activity	N/A
Thornton et al., 2021	Explicit in paper	1.1 Goal setting (behavior) 2.2 Feedback on behavior 2.3 Self-monitoring of behavior 7.1 Prompts/cues 10.3 Non-specific reward	Hypothesized	● Only a quarter of students reported wanting to use the app frequently, but provided positive feedback about behavior tracking, goal setting, reminders, and personalized feedback features of the app ● No consensus on whether competition would be helpful or detrimental	*
Whiteley et al., 2018	Inferred	1.2 Problem solving 3.1 Social support (unspecified) 3.2 Social support (practical) 5.1 Information about health consequences 5.2 Salience of consequences 10.3 Non-specific reward 12.1 Restructuring the physical environment	Hypothesized	● Interviews with youth indicated a desire for informational game content with comprehensive detail, future orientation, positive reinforcement, and that promote collaboration with healthcare providers and provided strategies	**
Whiteley et al., 2019	Inferred	1.2 Problem solving 3.1 Social support (unspecified) 3.2 Social support (practical) 5.1 Information about health consequences 5.2 Salience of consequences 8.7 Graded tasks 10.3 Non-specific reward	Hypothesized	● Similar findings to Whiteley et al. (2018) ● Interviews also indicated that participants wanted levels that become increasingly difficult (for a sense of accomplishment)	**
Baumel and Kane, 2018	Inferred	2.2 Feedback on behavior 10.3 Non-specific rewards	Inferred by reviewers	● Two items of the Therapeutic Persuasiveness domain scale are aligned with BCTs: rewards and ongoing feedback ● Both were significantly positively associated with mobile app user retention after 30 days (*r* = 0.26, *p* = 0.03 and *r* = 0.33, *p* = 0.01, respectively) ● Neither were significantly associated with usage time	N/A
Buman et al., 2016	Explicit in paper	**Sleep component:** 1.1 Goal setting (behavior) 1.2 Problem solving 2.2 Feedback on behavior 2.3 Self-monitoring of behavior 4.2 Information about antecedents 7.3 Reduce prompts/cues **Sedentary behavior component:** 2.2 Feedback on behavior 2.3 Self-monitoring of behavior 5.1 Information about health consequences 7.1 Prompts/cues 8.2 Behavior substitution **Exercise component:** 1.1 Goal setting (behavior) 1.2 Problem solving 2.2 Feedback on behavior 2.3 Self-monitoring of behavior 4.1 Instruction to perform the behavior 5.1 Information about health consequences 7.1 Prompts/cues	Inferred by reviewers	● Steady use of sleep and sedentary app components over intervention period (∼30min/week), but not exercise component - implies potential association between BCTs included in these components and engagement ● Participants disliked exclusion of “lighter intensity” exercise activities - implies importance of BCT 8.7 Graded tasks^c^	Some concerns (RoB)
Caon et al., 2022	Explicit in paper	7.1 Prompts/cues^b^ 10.3 Non-specific rewards^b^	Inferred by reviewers	● Just over 1/3 of participants (37.5% used app for more than 2 weeks) ● No direct association between engagement and BCTs included in app	**** *
Cueto et al., 2019	Explicit in paper	1.1 Goal setting (behavior) 1.2 Problem solving 1.3 Goal setting (outcome) 1.4 Action planning 1.5 Review behavior goal(s) 1.7 Review outcome goals 2.2 Feedback on behavior 2.3 Self-monitoring of behavior 2.5 Monitoring of outcomes of behavior 3.1 Social support (unspecified) 8.2 Behavior substitution 8.3 Habit formation	Inferred by reviewers	● High overall program retention (79.9%) implies that included BCTs associated with engagement	****
Davis and Ellis, 2019	Explicit in paper	**Noom Walk Pedometer:** 2.3 Self-monitoring of behavior^b^ 6.3 Information about others’ approval^b^ **MapMyFitness:** 1.1 Goal setting (behavior) 1.5 Review behavioral goals 2.3 Self-monitoring of behavior^b^ 2.4 Self-monitoring of outcomes of behavior 4.1 Instruction on how to perform the behavior 6.2 Social comparison 6.3 Information about others’ approval^b^ 7.1 Prompts/cues 8.7 Graded tasks 10.3 Non-specific reward	Inferred by reviewers	● Comparison of apps with different numbers of BCTs found the app with more BCTs had a significantly higher mean rating on the uMARS engagement subscale [*t*(81) = 6.71, *p* < 0.001, *g* = 1.15, 95% confidence interval (1.02, 1.87)] ● Implies that BCTs present only in MapMyFitness might contribute to engagement ● Potential confounding factors: apps could differ on items other than BCTs (e.g., usability, aesthetics)	Low RoB
DeSmet et al., 2019	Explicit in paper	2.2 Feedback on behavior 2.3 Self-monitoring of behavior 4.1 Instruction on how to perform behavior 5.1 Information on health consequences, self-monitoring	Inferred by reviewers	● Survey found that most preferred BCTs were: information on behavior health outcomes, obtaining insights into their healthy lifestyles, self-monitoring, feedback, instructions, or tips ● Lowest ranked were: social support and social comparison	***
Höchsmann et al., 2019	Explicit in paper	1.1 Goal setting (behavior) 1.4 Action planning 2.2 Feedback on behavior 7.1 Prompts/cues 10.3 Non-specific reward	Inferred by reviewers	● Intervention group had a significant improvement in intrinsic physical activity motivation (IMI total score) compared to control group (adjusted difference of 8.15 points, 95% CI 0.90–15.39; *p* = 0.03) ● Intervention group also had a significantly increase in the “interest/enjoyment” subscale (adjusted difference = 2.03 points, 95% CI 0.04–4.09; *p* = 0.049) ● Intervention group had a weekly average of 131.1 (SD 48.7) minutes of in-game walking and 15.3 (SD 24.6) minutes of strength training ● Implies potential positive association between included BCTs and engagement	Some concerns (RoB)
Nurmi et al., 2020	Explicit in paper	1.1 Goal setting (behavior) 1.3 Goal setting (outcome) 1.4 Action planning 1.5 Review behavior goal(s) 1.6 Discrepancy between current behavior and goal 1.7 Review outcome goals 2.2 Feedback on behavior 2.3 Self-monitoring of behavior 2.6 Biofeedback 3.1 Social support (unspecified - motivational interviewing) 10.4 Social reward 15.2 Mental rehearsal of successful performance 15.3 Focus on past success	Inferred by reviewers	● Interviews and theory identified key features relating to autonomy support and change talk ● Participants reported that features related to autonomy (eg. personalized goals, tailored feedback) and relatedness (motivational interviewing features) helped support motivation to engage	**** *
Valle et al., 2020	Inferred	1.1 Goal setting (behavior)^b^ 2.2 Feedback on behavior^b^ 2.3 Self-monitoring of behavior^b^ 3.1 Social support (unspecified)^b^ 7.1 Prompts/cues^b^	Inferred by reviewers	● No direct association evidenced between BCTs and engagement measures ● Past success appeared to be associated with increased engagement ● BCT 15.3 Focus on past success^c^ could potentially help support engagement, but possible that people who had greater past success were more committed for other reasons	***

^a^Overall RoB2 assessments are reported as “high,” “low,” or “some concerns” and overall MMAT assessments are reported as * (0–5); three studies (a content analysis and two development papers) could not be assessed and are noted as “N/A.”

^b^The authors deemed there was insufficient evidence of a potential association with engagement to justify including these BCTs in Table 7.

^c^These BCTs were not included in the apps evaluated, but were deemed by the authors to have potential associations with engagement based on the papers’ reported findings and included in Table 7.

**TABLE 7:** Associations between behaviour change techniques (BCTs) and engagement identified in the studies.

Evidence (# of studies in which BCT was associated with engagement)	Evidenced (*n* = 8)	Hypothesized (*n* = 11)	Inferred (*n* = 9)	Total (*n* = 28)
1.1 Goal setting (behavior)	5	6	6	17
1.2 Problem solving		3	2	5
1.3 Goal setting (outcome)		1	2	3
1.4 Action planning	3	2	3	8
1.5 Review behavior goal(s)		1	3	4
1.6 Discrepancy between current behavior and goal			1	1
2.2 Feedback on behavior	3	7	7	17
2.3 Self-monitoring of behavior	3	7	5	15
2.4 Self-monitoring of outcomes of behavior			1	1
2.5 Monitoring of outcomes of behavior			1	1
2.6 Biofeedback			1	1
3.1 Social support (unspecified)	5	5	3	13
3.2 Social support (practical)	2	4		6
3.3 Social support (emotional)		3		3
4.1 Instruction to perform the behavior		3	3	6
4.2 Information about antecedents		1	1	2
5.1 Information about health consequences	1	5	2	8
5.2 Salience of consequences		2		2
5.5 Anticipated regret	1			1
5.6 Information about emotional consequences	1	1		2
6.1 Demonstration of the behavior	1	1		2
6.2 Social comparison		3	1	4
7.1 Prompts/cues	3	6	4	13
7.3 Reduce prompts/cues			1	1
8.1 Behavioral practice/rehearsal		1		1
8.2 Behavior substitution			2	2
8.3 Habit formation	1	1	1	3
8.7 Graded tasks		1	2	3
9.1 Credible source		1		1
10.2 Material reward		2		2
10.3 Non-specific reward	2	7	3	12
10.4 Social reward			1	1
10.9 Self-reward	1			1
11.2 Reduce negative emotions		1		1
12.1 Restructuring the physical environment		1	1	2
12.5 Adding objects to the environment		1		1
14.2 Punishment		1		1
15.1 Written persuasion about capabilities		1		1
15.2 Mental rehearsal of successful performance			1	1
15.3 Focus on past success			1	1
Count of BCTs	14	29	26	40

The shading of the colours reflects the number of studies in which the BCT was associated with engagement (darker colours = more studies).

### 3.6. Exploratory analysis of engagement components

Only two studies explicitly discussed multi-faceted conceptualizations of engagement (Nurmi et al., 2020; Szinay et al., 2021). 21 studies included measures of engagement with sufficient description to code various components, most of which captured some form of behavioral engagement (16/21), either with the intervention (12/21) or with the target behavior (11/21). All of the studies that examined engagement with the intervention analyzed app use data, sometimes supplemented by self-report (5/12). Health behavior data was captured through self-report via app or questionnaire in all of the studies but one, which used the app to track step count (Höchsmann et al., 2019).

Around half of the studies captured affective (10/21) and cognitive (11/21) components of engagement. Although affective engagement was seldom a stated outcome, a couple common affective-related themes were identified in several qualitative studies, notably “motivation” and “fun” (Tang et al., 2015; Buman et al., 2016; Hales et al., 2016; Duff et al., 2018; D’Addario et al., 2020; Szinay et al., 2021; Thornton et al., 2021). Participants suggested that features providing social support (D’Addario et al., 2020; Szinay et al., 2021), feedback on behavior (D’Addario et al., 2020), prompts and cues (Duff et al., 2018), rewards (Hales et al., 2016; Szinay et al., 2021), and encouragement (Hales et al., 2016; Szinay et al., 2021) were motivating and that gamification, such as challenges and competition, was both motivating and fun (Tang et al., 2015; Hales et al., 2016; Szinay et al., 2021). Not all participants felt the apps needed to be fun, as that was not the reason they were engaging with them (Duff et al., 2018; D’Addario et al., 2020). Some studies also identified negative emotions (e.g., discouragement, guilt) associated with feedback that increased users’ awareness of their less healthy behaviors (Tang et al., 2015; Buman et al., 2016).

Various constructs related to affective engagement were also captured in various questionnaires: irritation (EQR User Engagement subscale), sense of empathizing from app (EQR Therapeutic Alliance subscale) inspiration/encouragement (EQR Therapeutic Persuasiveness subscale), fun and entertainment (uMARS engagement section), and visual appeal (EQR Visual Design subscale and uMARS aesthetics section) (Baumel and Kane, 2018; Davis and Ellis, 2019), “hedonic motivation” (UTAUT2) (Duff et al., 2018), and “interest/enjoyment” (IMI subscale) (Höchsmann et al., 2019).

Data related to cognitive engagement also came primarily from qualitative feedback (Tang et al., 2015; Buman et al., 2016; Hales et al., 2016; Duff et al., 2018; Whiteley et al., 2019; Nurmi et al., 2020; Szinay et al., 2021; Thornton et al., 2021), although a few questionnaires included at least one item relating to interest (EQR User Engagement subscale (Baumel and Kane, 2018), uMARS engagement section (Davis and Ellis, 2019), IMI “interest/enjoyment” subscale (Höchsmann et al., 2019), and the Client Satisfaction Questionnaire (Whiteley et al., 2019). Qualitative results identified a dichotomous association between cognitive effort and engagement; some feedback suggested that engagement would be negatively affected by a time-consuming app with a high cognitive load and that a basic, easy-to-use app was desirable (Szinay et al., 2021), while other findings indicated that lots of content could help sustain engagement and disliked apps that were too basic (Tang et al., 2015; Thornton et al., 2021).

### 3.7. Risk of bias and quality assessment

The risk of bias of the six randomized trials was evaluated using the Cochrane Collaboration RoB 2 tool (Higgins et al., 2011; The Cochrane Collaboration, 2021; Table 8 and Supplementary Appendix 6). The domains with the lowest risk of bias were “measurement of the outcome” (because most of the studies used objective measures of engagement) and “missing outcome data” (largely because engagement was the main outcome, so drop-out and attrition were data). Bias in the selection of the reported result could not be determined for the majority of studies, as pre-published protocols or registrations were not found.

**TABLE 8:** Risk of Bias 2 assessment of randomized studies.

References	Random-ization process	Period and carryover effects (crossover trials only)	Deviations from intended interventions (assignment)	Deviations from intended interventions (adherence)	Missing outcome data	Measurement of the outcome	Selection of the reported result	Overall bias
Buman et al., 2016	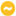	N/A	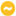	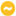	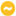	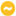	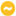	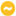
Davis and Ellis, 2019	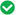	N/A	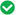	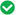	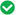	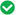	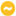	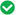
Du et al., 2016	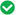	N/A	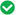	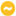	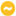	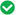	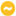	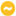
Höchsmann et al., 2019	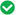	N/A	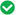	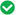	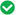	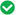	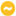	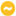
Nuijten et al., 2021	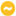	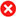	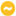	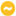	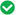	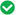	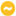	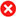
Nuijten et al., 2022	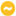	N/A	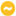	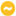	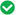	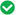	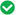	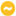

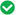
 = low risk, 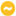 = some concerns, 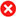 = high risk (Risk of Bias 2 (RoB 2) tool, n.d.; Higgins et al., 2011).

The Mixed-Methods Appraisal Tool (MMAT) (Hong et al., 2018) was used to assess the quality of the remaining studies (Supplementary Appendix 6). Of the study types, the qualitative studies met the most criteria; however, as the individual quality criteria are different for each study, a direct comparison is inappropriate. Common issues with the mixed-methods studies assessed were a lack of explicit justification for the mixed-methods design and a lack of detailed interpretation of the integration of results. For the six quantitative studies, half did not have participants representative of the target population. Across all of the study types, there was a common issue regarding the item about “clear research questions”; two-thirds of the studies (13/19) reported aims, but not research questions.

## 4. Discussion

### 4.1. Principal findings

In total, 28 studies that developed or examined mobile apps for health behavior change were examined to investigate how behaviour change techniques (BCTs) could influence different components of engagement. Although a variety of methods were used and various components of engagement could be identified in the data captured, most studies did not purposefully examine these different components. Different measures tended to capture different components: affective and cognitive engagement were primarily captured with qualitative methods, micro behavioral engagement with app use data, and macro behavioral engagement with self-report, questionnaires, and app tracking. This emphasizes the importance of mixed methods to fully assess user engagement. By combining study-reported evidence with our coding of BCTs and engagement, we identified patterns of evidence that suggest the potential of 6 BCTs to support engagement with digital health interventions: social support, goal setting, feedback, prompts/cues, self-monitoring, and rewards. A lack of explicit reporting of BCTs and preplanned analyses of engagement components limited our ability to identify potential associations between BCTs and specific components of engagement.

### 4.2. Comparisons with existing literature

The BCTs identified as having evidence of associations with engagement are in line with previous findings. The mixed influence of social support on engagement has also been observed by previous research (Tong and Laranjo, 2018; Szinay et al., 2020) and could potentially be explained by a difference between hypothetical preferences and practical experiences (Szinay et al., 2020). The implementation of the social support BCT could also be a factor; the studies that found a positive influence of social support on engagement were team-based (Du et al., 2016; Nuijten et al., 2021), provided practical social support, such as connection with healthcare professionals, or offered optional opportunities to interact with other users to get peer support, compete, or be held accountable to their goals (Tang et al., 2015; Szinay et al., 2021). Mixed or negative influences were associated with social comparison and competition (D’Addario et al., 2020).

### 4.3. Strengths and limitations

The broad scope is both a strength and a limitation; the inclusive search strategy reduced the likelihood of missing relevant studies, but necessitated automated screening. To mitigate the risk of screening out relevant studies, we used a set of six articles identified in preliminary searches and determined to be eligible to check the screening. After each pass in EndNote, if any of the six studies were missing, the search terms were revised and the pass re-run to ensure we were not incorrectly eliminating relevant papers. Although the search terms were developed with a second author, the review was not independently executed by two reviewers, which would have reduced the risk of bias in the analysis and increased the credibility of the conclusions. It was not possible to have a second reviewer due to time and resource constraints.

The diversity of outcomes examined by the studies prohibited a quantitative meta-analysis to examine effect. Additionally, the studies examined a wide variety of health apps, aimed at various populations and behaviors. In including this variety of studies, the review is limited in its ability to explore potential context-specific associations. Contextual factors such as behavior, age, culture, app type, and outcomes could influence engagement in ways that it was not possible to capture with the current sample. The study was also limited to apps that had been evaluated in scientific investigations; this method risks excluding popular publicly-available apps that have not been previously evaluated. A review of the BCTs included in such apps and how they are associated with engagement could provide additional insights to inform app design.

This study instead contributes to the literature by analyzing the body of evidence to hypothesize associations between BCTs and engagement for future testing. The analysis was limited by the available reported data and descriptions of the studies’ apps and measures. The lack of explicit analyses of engagement components in the included studies meant the review relied on author coding and inference to identify potential associations. As such, this review represents a starting point for further investigation of engagement and an identification of current limitations in digital health methodology and reporting.

### 4.4. Future research and recommendations

One of the challenges of our analysis was that there were no clear and comprehensive definitions of affective, cognitive, and behavioral engagement in the literature. Constructs commonly associated with affective engagement included motivation, positive and negative feelings, and sometimes aesthetic pleasure, while cognitive engagement was described using terms such as interest, attention, thought, and challenge (O’Brien, 2016; Perski et al., 2017; Kelders et al., 2020). Recent discussions in the literature have highlighted the importance of using mixed-methods designs to enable these elements to be assessed (Yardley et al., 2016; Perski et al., 2017; Short et al., 2018), which is supported by our findings. As more studies intentionally examine the different components of engagement and how they influence each other, our understanding of how to use particular BCTs to provide tailored support for different individuals, in different contexts, and at different times in their process of engagement with the intervention will improve.

There was not sufficient evidence available in the included studies to posit associations between BCTs and specific components of engagement. However, the exploratory analysis enables some hypotheses to be suggested for further investigation. Several of the BCTs with potential associations with engagement - goal setting, self-monitoring, feedback, social support, rewards, and prompts and cues - were identified as being “motivating” and “fun” for users. This evidence could indicate associations between these BCTs and affective and cognitive engagement, although further context about how and why they were motivating it would be needed to determine whether the associations were primarily affective, cognitive, or both. A theoretical examination of those components compared to the Behaviour Change Technique (BCT) taxonomy can provide some additional insight.

The Human Behavior Change Project’s Theory and Techniques Tool maps the BCT taxonomy with mechanisms of action (MoA) (Connell Bohlen et al., 2018; Carey et al., 2019; Johnston et al., 2021). A couple of the MoAs associated with the BCTs clearly relate to a component of engagement; for example, the definitions of MoAs with links to goal setting (intention and goals) are theoretically similar to the concept of cognitive engagement as they relate to “conscious decisions” and “mental representations,” as is one of the MoAs linked with prompts/cues (memory, attention, and decision processes). This suggests that, for these two BCTs in particular, their impact on motivation is more likely to be related to cognitive than affective engagement. However, many of the linked MoAs do not provide any additional clarity about how the BCTs relate to different components of engagement. For instance, one of the MoAs for self-monitoring (behavioral regulation) is defined as “behavioral, cognitive and/or emotional skills for managing or changing behavior.” Likewise, the MoA environmental context/resources, which is linked with social support and prompts/cues, is defined as “aspects of a person’s situation or environment that discourage or encourage the behavior,” which could conceivably include cognitive, affective, or behavioral aspects. In digital health, these aspects can include the availability, accessibility, and interactivity of an app (potentially affecting behavioral engagement), the tone of the app (potentially affecting affective engagement), and the personalization to a users’ needs (potentially affecting cognitive engagement) (Szinay et al., 2020, 2021).

Recent studies have identified a lack of justification and guidance for the operationalization of BCTs (Fulton et al., 2018; Lemke and de Vries, 2021). The evidence indicates that the way BCTs such as social support are incorporated into interventions could affect how they are associated with engagement. It will be important to examine if there are certain operationalizations that better support engagement or whether its influence is related primarily to individual characteristics. Understanding when and how they have a positive or negative impact would enable interventions to be tailored to the specific context in which they will be implemented or dynamically to individuals, to emphasize specific BCTs and app features that they are most likely to respond best to.

For app developers, the key takeaways from the evidence gathered in this review is that the inclusion of BCTs such as goal setting, feedback, prompts/cues, self-monitoring, and reward could help support users’ engagement with mobile health apps. Social support also has the potential to support engagement, but can have unintended negative effects, depending on how it is incorporated. Based on the studies reviewed, our recommendation for developers considering incorporating social support is to carefully consider how it will be operationalized and to make that feature optional for users. Practical social support, such as linking patients with healthcare providers, is likely to support engagement and team-based competition seems to be less likely than individual competition to generate negative emotions in users. In view of app design, there appears to be a balance needed to obtain the optimal level of cognitive load. Data from participants indicated a preference for a reduced cognitive load - limited complexity, less data input, limited number of features, not time-consuming (Abuhamdeh et al., 2015; Szinay et al., 2021) - while other data and motivation theory suggests that the app needed to be interesting enough to keep users’ attention (Tang et al., 2015; Thornton et al., 2021) and provide the right level of challenge to achieve optimal motivation (Abuhamdeh et al., 2015). Therefore, apps should be easy to learn and efficient to navigate (Wei et al., 2020), but contain sufficient or varying content to sustain users’ interest. Finally, it is likely to be important for engagement to choose BCTs and other app features carefully to fit the context and the aim of the intervention, rather than incorporating as many BCTs as possible.

## 5. Conclusion

The purpose of this systematic review was to incorporate theoretical conceptualizations of engagement and synthesize evidence of how mobile health apps and their BCTs are associated with different components of user engagement. Six BCTs were identified as having the most evidence of a potential association with engagement with digital behavior change interventions: goal setting, self-monitoring of behavior, feedback on behavior, prompts/cues, rewards, and social support. An exploratory analysis of the engagement components identified key areas for future research, including further theoretical clarity of engagement components, how less researched BCTs might be theoretically linked to engagement components, and how BCTs can be operationalized in app design to best support affective, cognitive, and behavioral engagement. Recommendations for app development deriving from the findings are the inclusion and further investigation of the 6 BCTs identified to support engagement and the importance of identifying the right level of cognitive involvement for the target population to keep the app interesting without being a burden. This review contributes to the body of evidence for the influence of specific BCTs on engagement by identifying a gap between theoretical conceptualizations of engagement and its evaluation in mobile health apps and hypothesizing potential associations for future empirical investigation. Greater clarity in the definitions of different components of engagement, and further development and incorporation of means of measuring specific types of engagement, could enable future research to examine how different components of engagement are related to specific BCTs and behavioral and health outcomes. This knowledge could inform the design and tailoring of mobile health apps to improve engagement with the intervention and target behaviors.

## Data availability statement

The original contributions presented in this study are included in the article/Supplementary material, further inquiries can be directed to the corresponding author.

## Author contributions

MM-I conceived the topic, drafted the protocol, conducted the searches, screening, data extraction and analysis, and wrote the first draft of the manuscript. SH and JA contributed to the revisions. EM performed the final review and supervised the execution of the review. All authors contributed to the article and approved the submitted version.

## Supplementary material

Supplementary Appendix 1 PRISMA checklist.

Supplementary Appendix 2 Search results by database.

Supplementary Appendix 3 EndNote screening.

Supplementary Appendix 4 Engagement components codebook.

Supplementary Appendix 5 Study characteristics.

Supplementary Appendix 6 Risk of bias and quality assessment.
