# Supplementary material for: Potential associations between behavior change techniques and engagement with mobile health apps: a systematic review
Source: Front Psychol. 2023 Sep 18;14:1227443. doi: 10.3389/fpsyg.2023.1227443 (PMC10545861; doi:10.3389/fpsyg.2023.1227443)
Supplement: Supplementary Appendix 2 — Search results by database. [file Table_2.docx]

## **Appendix 2. Search results by database**

| **Database** | **Search string** | **References** |
| --- | --- | --- |
| PubMed | (((Behavior Control OR Psychological Theory[MeSH Terms]) OR ("behavior change techniques"[Title/Abstract] or "behavior change techniques"[Title/Abstract] or "BCT"[Title/Abstract] or "behavior change technique"[Title/Abstract] or "behavior change technique"[Title/Abstract] or "behavioral change strategies"[Title/Abstract] or "behavioral change strategies"[Title/Abstract] or "behavior change wheel"[Title/Abstract] or "behavior change wheel"[Title/Abstract] or "behavioral theory"[Title/Abstract] or "behavioral theory"[Title/Abstract] or "behavior change theory"[Title/Abstract] or "behavior change theory"[Title/Abstract] or "health behavior change"[Title/Abstract] or "behavior change"[Title/Abstract] or "behavior change"[Title/Abstract] or "digital behavior change intervention"[Title/Abstract] or "digital behavior change intervention"[Title/Abstract] or "DBCI"[Title/Abstract] or "behavior change intervention"[Title/Abstract] )) AND ((Telemedicine OR Mobile Applications OR Internet-Based Intervention[MeSH Terms]) OR ("mHealth"[Title/Abstract] OR "eHealth"[Title/Abstract] OR "mobile health"[Title/Abstract] OR "eHealth"[Title/Abstract] OR telehealth[Title/Abstract] OR mobile[Title/Abstract] OR phone[Title/Abstract] OR smartphone[Title/Abstract] OR cell[Title/Abstract] OR digital[Title/Abstract] OR "app"[Title/Abstract] OR "apps"[Title/Abstract] OR application*[Title/Abstract] OR digital[Title/Abstract] OR web[Title/Abstract] OR internet[Title/Abstract] OR online[Title/Abstract] OR technology[Title/Abstract] ))) AND ((Treatment Adherence and Compliance OR Patient Participation OR Patient Compliance[MeSH Terms]) OR (Engag*[Title/Abstract] OR "user engagement"[Title/Abstract] OR immersion[Title/Abstract] OR flow[Title/Abstract] OR involvement[Title/Abstract] OR presence[Title/Abstract] OR adherence[Title/Abstract] OR attrition compliance[Title/Abstract] OR maintenance[Title/Abstract] OR acceptability[Title/Abstract] OR satisfaction[Title/Abstract])) | 12,397 |
| Embase (Ovid) | ((patient compliance/ or patient participation/) OR ((Engag* or "user engagement" or immersion or flow or involvement or presence or adherence or attrition compliance or maintenance or acceptability or satisfaction).ti,ab)) AND ((telemedicine/ or mobile application/ or internet-based intervention/) OR (("mHealth" or "eHealth" or "mobile health" or "eHealth" or telehealth or mobile or phone or smartphone or cell or digital or "app" or "apps" or application* or digital or web or internet).ti,ab)) AND ((behavior control/ or psychological theory/) OR (("behaviour change techniques" or "behavior change techniques" or "BCT" or "behaviour change technique" or "behavior change technique" or "behavioral change strategies" or "behavioural change strategies" or "behaviour change wheel" or "behavior change wheel" or "behavioural theory" or "behavioral theory" or "behaviour change theory" or "behavior change theory" or "health behaviour change" or "behaviour change" or "behavior change" or "digital behaviour change intervention" or "digital behavior change intervention" or "DBCI" or "behaviour change intervention").ti,ab)) | 2,565 |
| CINAHL^a^ | (MH ( Treatment Adherence and Compliance OR Patient Participation OR Patient Compliance ) OR AB ( Engag* OR “user engagement” OR immersion OR flow OR involvement OR presence OR adherence OR attrition compliance OR maintenance OR acceptability OR satisfaction )) AND (MH ( Telemedicine OR Mobile Applications OR Internet-Based Intervention ) OR AB ( “mHealth” OR “eHealth” OR “mobile health” OR “eHealth” OR telehealth OR mobile OR phone OR smartphone OR cell OR digital OR “app” OR “apps” OR application* OR digital OR web OR internet )) AND (MH ( Behavior Control OR Psychological Theory ) OR AB ( “behavior change techniques” or “behavior change techniques” or “BCT” or “behavior change technique” or “behavior change technique” or “behavioral change strategies” or “behavioral change strategies” or “behavior change wheel” or “behavior change wheel” or “behavioral theory” or “behavioral theory” or “behavior change theory” or “behavior change theory” or “health behavior change” or “behavior change” or “behavior change” or “digital behavior change intervention” or “digital behavior change intervention” or “DBCI” or “behavior change intervention” )) | 672 |
| APA PsycArticles^a^ | (mainsubject(Treatment Adherence and Compliance OR Patient Participation OR Patient Compliance) OR ab(Engag* OR “user engagement” OR immersion OR flow OR involvement OR presence OR adherence OR attrition compliance OR maintenance OR acceptability OR satisfaction)) AND (mainsubject(Telemedicine OR Mobile Applications OR Internet-Based Intervention) OR ab(“mHealth” OR “eHealth” OR “mobile health” OR “eHealth” OR telehealth OR mobile OR phone OR smartphone OR cell OR digital OR “app” OR “apps” OR application* OR digital OR web OR internet)) AND (mainsubject(Behavior Control OR Psychological Theory) OR ab(“behavior change techniques” or “behavior change techniques” or “BCT” or “behavior change technique” or “behavior change technique” or “behavioral change strategies” or “behavioral change strategies” or “behavior change wheel” or “behavior change wheel” or “behavioral theory” or “behavioral theory” or “behavior change theory” or “behavior change theory” or “health behavior change” or “behavior change” or “behavior change” or “digital behavior change intervention” or “digital behavior change intervention” or “DBCI” or “behavior change intervention”)) | 66 |
| Science Direct^b^ | ("engagement") AND ("mobile" OR "digital" OR "mHealth") AND ("BCT" OR "behavior theory" OR "behavioral theory" OR "behavior change intervention") | 1,291 |
| Cochrane Library^c^ (Trials) | (Engag* OR “user engagement” OR immersion OR flow OR involvement OR presence OR adherence OR attrition compliance OR maintenance OR acceptability OR satisfaction) AND (mHealth OR eHealth OR “mobile health” OR telehealth OR mobile OR phone OR smartphone OR cell OR digital OR “app” OR “apps” OR application* OR digital OR web OR internet) AND (“behavior change techniques” or “behavior change techniques” or “BCT” or “behavior change technique” or “behavior change technique” or “behavioral change strategies” or “behavioral change strategies” or “behavior change wheel” or “behavior change wheel” or “behavioral theory” or “behavioral theory” or “behavior change theory” or “behavior change theory” or “health behavior change” or “behavior change” or “behavior change” or “digital behavior change intervention” or “digital behavior change intervention” or “DBCI” or “behavior change intervention”) | 1,779 |
| Web of Science^c,d^ | (Engag* OR “user engagement” OR immersion OR flow OR involvement OR presence OR adherence OR attrition compliance OR maintenance OR acceptability OR satisfaction) AND ("mHealth” OR “eHealth” OR “mobile health” OR “eHealth” OR telehealth OR mobile OR phone OR smartphone OR cell OR digital OR “app” OR “apps” OR application* OR digital OR web OR internet) AND (“behavior change techniques” or “behavior change techniques” or “BCT” or “behavior change technique” or “behavior change technique” or “behavioral change strategies” or “behavioral change strategies” or “behavior change wheel” or “behavior change wheel” or “behavioral theory” or “behavioral theory” or “behavior change theory” or “behavior change theory” or “health behavior change” or “behavior change” or “behavior change” or “digital behavior change intervention” or “digital behavior change intervention” or “DBCI” or “behavior change intervention”) | 2,415 |
| Total |  | 21,185 |
| Total | *(with 3245 duplicates removed by EndNote)* | 17,940 |

^a^CINAHL and APA PsycArticles do not have a Title/Abstract option, so the search was limited to abstracts

^b^Science Direct only allows 8 Boolean Operators to be used in a search, so key terms from each theme were selected and used in the search

^c^Cochrane Library and Web of Science do not use MeSH terms, so only keywords were searched

^d^Web of Science does not have a Title/Abstract search limiter, so ‘Topic’ was used (searches Title, Abstract, author keywords, and Keywords Plus)
