# Supplementary material for: Potential associations between behavior change techniques and engagement with mobile health apps: a systematic review
Source: Front Psychol. 2023 Sep 18;14:1227443. doi: 10.3389/fpsyg.2023.1227443 (PMC10545861; doi:10.3389/fpsyg.2023.1227443)
Supplement: Supplementary Appendix 3 — EndNote screening. [file Table_3.docx]

## **Appendix 3. EndNote screening**

| **Pass^a^** | **Search string** | **# of references remaining** | **Test articles present?** |
| --- | --- | --- | --- |
| 1 | Year = GREATER OR EQUAL TO 2012 | 13,710 | Yes |
| 2 | Title = NOT (review OR protocol OR chapter OR congress OR conference OR poster OR abstracts) | 9,144 | Yes |
| 3^b^ | Any Field = (behaviour change OR behavior change OR behaviour theory OR behavioural theory OR behavior theory OR behavioral theory OR social cognitive theory OR planned behavio* OR transtheoretical OR IMB) | 4,061 | Yes |
| 4 | Any Field = (digital OR mobile OR “app” OR “apps” OR “application” OR internet OR web OR phone OR telehealth OR mHealth) | 3,114 | Yes |
| 5 | Any Field = (engag* OR adher* OR immers* OR involve* OR adopt OR maint* OR sustain* OR app us* OR acceptab*) | 2,608 | Yes |
| 6 | Title OR Abstract = (behavio*) | 2,413 | Yes |
| 7 | Abstract = (engage OR adhere OR app us* OR acceptability) | 2,018 | Yes |
| 8 | Abstract = (smartphone OR phone OR mobile OR cell OR mHealth OR iphone OR android OR tablet OR “app” OR “apps”) | 1,171 | Yes |
| 9 | Title = (behav* OR engag* OR adher*) | 420 | Yes |
| 10 | Any Field = NOT (text message OR SMS) | 322 | Yes |

^a^Each pass was conducted on the subset of studies retrieved in the previous pass

^b^Six articles previously identified as relevant were used throughout the screening process to ensure that search terms were not unintentionally eliminating relevant articles

^c^Four specific theories that were not captured with the general ‘behaviour theory’ terms were included in this pass based on a scoping review which identified theories of behaviour change. These were the four that were cited at least 10 times in the included articles.
