# Supplementary material for: Potential associations between behavior change techniques and engagement with mobile health apps: a systematic review
Source: Front Psychol. 2023 Sep 18;14:1227443. doi: 10.3389/fpsyg.2023.1227443 (PMC10545861; doi:10.3389/fpsyg.2023.1227443)
Supplement: Supplementary Appendix 4 — Engagement components codebook. [file Table_4.docx]

## **Appendix 4. Engagement components codebook**

- **Affective**: motivation, positive and negative feelings (e.g. enjoyment), sensory pleasure of UX [(O’Brien, 2016; Perski et al., 2017; Kelders et al., 2020)](https://paperpile.com/c/7FRPZX/0Pq6+7h74+yGJP)
- **Cognitive**: challenge, interest, thought, attention [(O’Brien, 2016; Perski et al., 2017; Kelders et al., 2020)](https://paperpile.com/c/7FRPZX/0Pq6+7h74+yGJP)
- **Behavioural**: interactivity, usage (amount, duration, frequency, etc.) [(O’Brien, 2016; Perski et al., 2017; Kelders et al., 2020)](https://paperpile.com/c/7FRPZX/0Pq6+7h74+yGJP)
- **Micro:** engagement with the mobile health app itself [(Yardley et al., 2016)](https://paperpile.com/c/7FRPZX/lxxWF)
  - **UX**: “User interactions with the DBCI features and the context in which those interactions happen” [(Cole-Lewis et al., 2019)](https://paperpile.com/c/7FRPZX/S9o99)
  - **BCTs**: “Interactions with behaviour change intervention components/active ingredients specifically designed to influence the behavioural determinants which in turn influence the health behaviours” [(Cole-Lewis et al., 2019)](https://paperpile.com/c/7FRPZX/S9o99)
  - **Both**
  - **Unspecified**
- **Macro: “**engagement and identification with the wider intervention goals” [(Yardley et al., 2016)](https://paperpile.com/c/7FRPZX/lxxWF)
  - **Behaviour**: engagement with the targeted health behaviour [(Cole-Lewis et al., 2019)](https://paperpile.com/c/7FRPZX/S9o99)
